# Energetics of optimal undulatory swimming organisms

**DOI:** 10.1371/journal.pcbi.1007387

**Published:** 2019-10-31

**Authors:** Grgur Tokić, Dick K. P. Yue

**Affiliations:** Department of Mechanical Engineering, Massachusetts Institute of Technology, Cambridge, Massachusetts, United States of America; Westfalische Wilhelms-Universitat Munster, GERMANY

## Abstract

Energy consumption is one of the primary considerations in animal locomotion. In swimming locomotion, a number of questions related to swimming energetics of an organism and how the energetic quantities scale with body size remain open, largely due to the difficulties with modeling and measuring the power production and consumption. Based on a comprehensive theoretical framework that incorporates cyclic muscle behavior, structural dynamics and swimming hydrodynamics, we perform extensive computational simulations and show that many of the outstanding problems in swimming energetics can be explained by considering the coupling between hydrodynamics and muscle contraction characteristics, as well as the trade-offs between the conflicting performance goals of sustained swimming speed *U* and cost of transport COT. Our results lead to three main conclusions: (1) in contrast to previous hypotheses, achieving optimal values of *U* and COT is independent of producing maximal power or efficiency; (2) muscle efficiency in swimming, in contrast to that in flying or running, decreases with increasing body size, consistent with muscle contraction characteristics; (3) the long-standing problem of two disparate patterns of longitudinal power output distributions in swimming fish can be reconciled by relating the two patterns to *U*-optimal or COT-optimal swimmers, respectively. We also provide further evidence that the use of tendons in caudal regions is beneficial from an energetic perspective. Our conclusions explain and unify many existing observations and are supported by computational data covering nine orders of magnitude in body size.

## Author summary

Fish-like undulating swimming exhibits remarkable speeds, efficiencies and versatility over many orders of magnitude in body size. Using theoretical considerations from first principles coupled with a novel muscle-hydrodynamic model, and extensive computations, we answer a number of outstanding controversies regarding swimming energetics. We find that maximizing swimming speed or minimizing cost of transport COT (energy consumed per body mass per distance traveled) do not, in general, correspond to optimal muscle or total efficiency (power produced as a fraction of power consumed). These efficiencies are found to decrease with the size of the swimmer, in surprising contrast to runners or fliers who are generally more efficient with increasing body size. For all these different modes of locomotion, it is COT that always decreases with body mass, and it is optimizing for COT (and not efficiency) that provides evolutionary drive for greater body sizes. Our conclusions explain and unify existing observations and are supported by our computational data covering nine orders of magnitude in body size.

## Introduction

From the very beginning of the study of animal swimming, the problem of swimming energetics has generated a lot of interest. The problem was brought to the forefront by Gray’s paradox [1]—the notion that dolphin’s muscles should produce seven times more power per unit mass than other types of mammalian muscles. This remained a long-standing controversy, but has finally been found as flawed [2].

Much less is known about the energetics of swimming than about its kinematics due to the inherent complexity of measuring energetic quantities in swimming animals. For example, empirical measurements of metabolic power consumption, one of the most important quantities characterizing locomotion in general, are usually obtained in an indirect way by measuring the oxygen consumption during swimming in a respirometer [3–6]. These experiments provide us with the knowledge on how the total consumption (and indirectly the swimming efficiency [3]) depends on the body size and speed, but not on how the consumption is distributed along a swimming body.

Unlike these efforts aimed at obtaining the overall power consumption, experimental efforts related to mechanical power have been focused on obtaining the longitudinal pattern of its production based on *in vivo* measurements of neural signals. One approach to obtaining the power output from a neural stimulus is to employ the work-loop technique [6–8], which is based on the assumption that an isolated bundle of muscle fibers produces the same amount of power as the one which is an integral part of a body, if activated and stretched in an identical way [9, 10]. A different approach is to use a mathematical model of muscle fiber response to predict the power output based on the measurements of EMG activation and the strain cycle [11, 12]. The results obtained from these two approaches, however, appeared conflicting [13–16]—some indicated that most of the power was produced anteriorly, while others indicated that most of it was produced posteriorly. This disparity has not been resolved yet.

These examples of long-standing controversies illustrate one of the core problems in swimming energetics [6, 17]—a lack of a comprehensive theoretical model that could satisfactorily address all the relevant physics, from muscle activation to the hydrodynamics of swimming, and, thus, provide an understanding of the underlying phenomena. As a result of this lack of theoretical understanding, further important questions remain unresolved. For example, the muscle efficiency in swimming, in contrast to that in flying or running, does not increase with the body size, and a satisfactory explanation is still missing [17]. At an organism level, it had been assumed that muscle fibers in fish operate at maximum possible power levels during swimming at maximum sustained speeds [3, 8, 18]; this premise was called into question by later experiments [6].

Current modeling efforts predominantly focus either solely on the hydrodynamics of swimming of certain species [19–22], or on modeling muscle activation and the transfer of forces to the fluid [23–29]. A notable drawback of all these models is that they cannot capture the metabolic power consumption, which is of central importance for understanding fitness. Furthermore, as they do not capture all the physics relevant to the mechanics of swimming, they are not suitable for studying undulatory swimmers of general shape and motion that are still constrained by the mechanical properties of tissues (e.g. muscles) and by the physics of the surrounding flow. In recent years, we developed a comprehensive model of sustained undulatory swimming based on a coupled consideration of muscle behavior during periodic contractions, structural dynamics and hydrodynamics [30]. The key ingredient here is the introduction of a muscle model that enables us to obtain both the mechanical power production and the previously intractable metabolic power consumption along the body from the knowledge of the required muscle contractive forces and velocities. With this model in place, we can obtain the full picture of swimming energetics from body and motion characteristics alone.

In this paper, we conduct a theoretical and an extensive computational analysis of the energetics of optimal undulatory swimmers based on our above-mentioned coupled model [30]. The swimmers are optimized with respect to two conflicting performance measures of arguably crucial importance [2]—achieving the maximum steady sustained swimming speed *U* and the minimum cost of transport COT. We present detailed energetic data of optimal model swimmers across nine orders of magnitude in size and discuss both the integrated values and the longitudinal distributions of the crucial energetic quantities (powers, efficiencies). We find that our predictions match the empirically observed data from swimming fish, and we offer explanations for the aforementioned controversies in swimming energetics. We show that they can be reconciled by considering the coupled interaction between the muscle behavior and hydrodynamic forces and by considering the trade-offs between *U*-optimal and COT-optimal swimmers. These results and analyses complement those presented in our previous study [30].

### Model of an undulatory swimmer

We study sustained straight-line undulatory swimming, where an organism passes a muscle-produced wave of curvature down its body and propels itself using the hydrodynamic forces exerted on the body as a reaction to the undulatory motion. Intermittent undulatory motion, such as burst-and-coast swimming, is not considered in this work.

**Fig 1:**
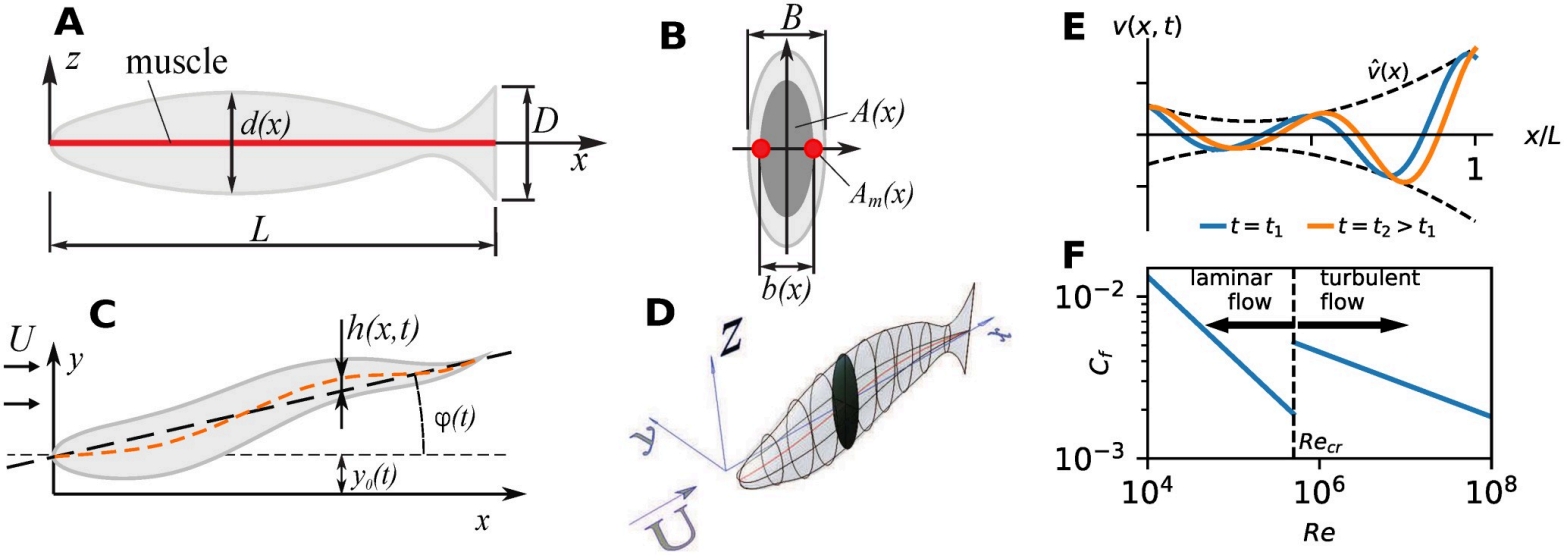
Description of body shape and motion. (A) Lateral view of a swimmer (arbitrary shape) and idealized muscle layout (red line). (B) Body cross section (area *A*(*x*)) and muscle cross section (area *A*_*m*_(*x*)) on each side of the body (red). (C) Dorsal view of the motion kinematics. (D) Three-dimensional view of a body with a cross-section highlighted. (E) Instantaneous contraction velocity *v*(*x*, *t*) along the body and its envelope $\hat{v}\left(x\right)$. (F) Friction drag coefficient *C*_*f*_. The critical Reynolds number is *Re*_*cr*_ = 5 × 10^5^. For the exact expression for *C*_*f*_ and the shape-dependent drag coefficient *C*_*D*_, see Eq (8).

We consider an idealized swimmer of mass *m* and arbitrary three-dimensional shape characterized by its body length *L*, tail height *D* and body width *B*, Fig 1A and 1B. We assume that the body is symmetric with respect to the horizontal and vertical planes, with elliptical cross sections of area *A*(*x*). The height and width distributions are denoted by *d*(*x*) and *b*(*x*), respectively.

The locomotory muscle is arranged into a thin axial strip of muscle fibers located superficially along the horizontal symmetry plane on each side of the body, i.e. located 1/2*b*(*x*) away from the body symmetry plane, Fig 1A and 1B. The cross section area *A*_*m*_(*x*) of the muscle on one side of the body is a small portion *μ*_0_ of the body cross-section *A*(*x*) (*A*_*m*_(*x*) = 0.5*μ*_0_*A*(*x*), *μ*_0_ ≪ 1); the rest of the body is considered passive and visco-elastic. The mass of the muscle on one side of the body *m*_*M*_ = 0.5*μ*_0_*m* is also equal to the maximum possible mass of activated muscle fibers since the fibers on only one side of the body are active at any time and any longitudinal location.

We describe the undulatory motion of the body neutral line *h*(*x*, *t*) using a single time harmonic [4, 14]:

$$\begin{array}{c} h\left(x,t\right)=r\left(x\right)\;\text{cos}\left(2\pi x/\lambda_{b}-\omega t\right)\;, \end{array}$$ (1)

where *ω* is the angular frequency of tail beat, *r*(*x*) is the deformation envelope and λ_*b*_ the wavelength of the body undulation. The instantaneous contraction speed *v*(*x*, *t*) of superficial muscle fibers is given by

$$\begin{array}{c} v\left(x,t\right)=\pm \frac{1}{2}b\left(x\right)\frac{\partial }{\partial t}(\frac{\partial^{2}h}{\partial x^{2}})\;, \end{array}$$ (2)

where the plus(minus) sign corresponds to the fibers on the right(left) side of the body being active. For periodic motion described by (1), the amplitude of *v* is directly proportional to the tail-beat frequency *ω*.

Describing the most important phenomena relevant for the mechanical and energetic aspects of swimming requires a comprehensive model that captures the physics from the force creation and energy consumption in the muscles to the generation of thrust force. We introduced such a comprehensive model in [30], based on simple, fast models that are valid for a problem of such general nature and its vast computational scope. This comprehensive model can be divided into three parts: (i) a hydrodynamic model that provides the forces exerted on the body during swimming and determines the swimming speed *U*, (ii) a structural model that determines the bending moment *M*(*x*, *t*) that is necessary to sustain the undulatory motion (see (9)), and (iii) a muscle model that determines the feasibility and the energy cost of such motion. In this study we use the comprehensive model introduced in [30] and provide a further analysis of the muscle submodel. The main aspects of the hydrodynamic and structural models are briefly explained in the Models section; for more details, see [30].

We consider large Reynolds number *Re* ≳ 10^4^ flows only (*Re* = *UL*/*ν*, *ν* is the kinematic viscosity of the fluid). To model the flow around a swimmer, we use the simple Lighthill’s slender-body potential flow model [31], which enables us to model swimmers of diverse shapes over a wide range of large Reynolds numbers. For the justification of this model, see Models section. It is important to note that the modeled hydrodynamic drag on a swimmer exhibits a discrete jump in value when the flow around the swimmer transitions from a laminar to a turbulent one. This occurs when the Reynolds number *Re* reaches a critical value *Re*_*cr*_. The resulting friction drag coefficient *C*_*f*_ is modeled using an empirical formula (Eq (8)), Fig 1F. While the empirical formula presents a significant simplification, the physics it captures is present in reality—namely, the flow around a swimming organism, and the associated power required to support it, changes drastically as the flow transitions from laminar to turbulent.

In the following, we introduce our muscle model in more detail.

### Muscle performance in oscillatory contraction

The majority of muscle models are based on the given timing of a neural stimulus [11, 27, 28, 32], which can usually be obtained from electromyographic (EMG) and sonomicrometry recordings. While the response of a muscle fiber to a neural stimulus can be modeled relatively well, the downside of modeling the behavior of the entire muscle based on the timing of the stimulus is that the fraction of the activated muscle fibers at any location, and, hence, the resultant force and power, cannot be determined solely from it. Rather, this approach results in modeling the maximum *potential* for producing force or power [13]. The same downside exists for the results obtained from the work-loop technique [15].

**Fig 2:**
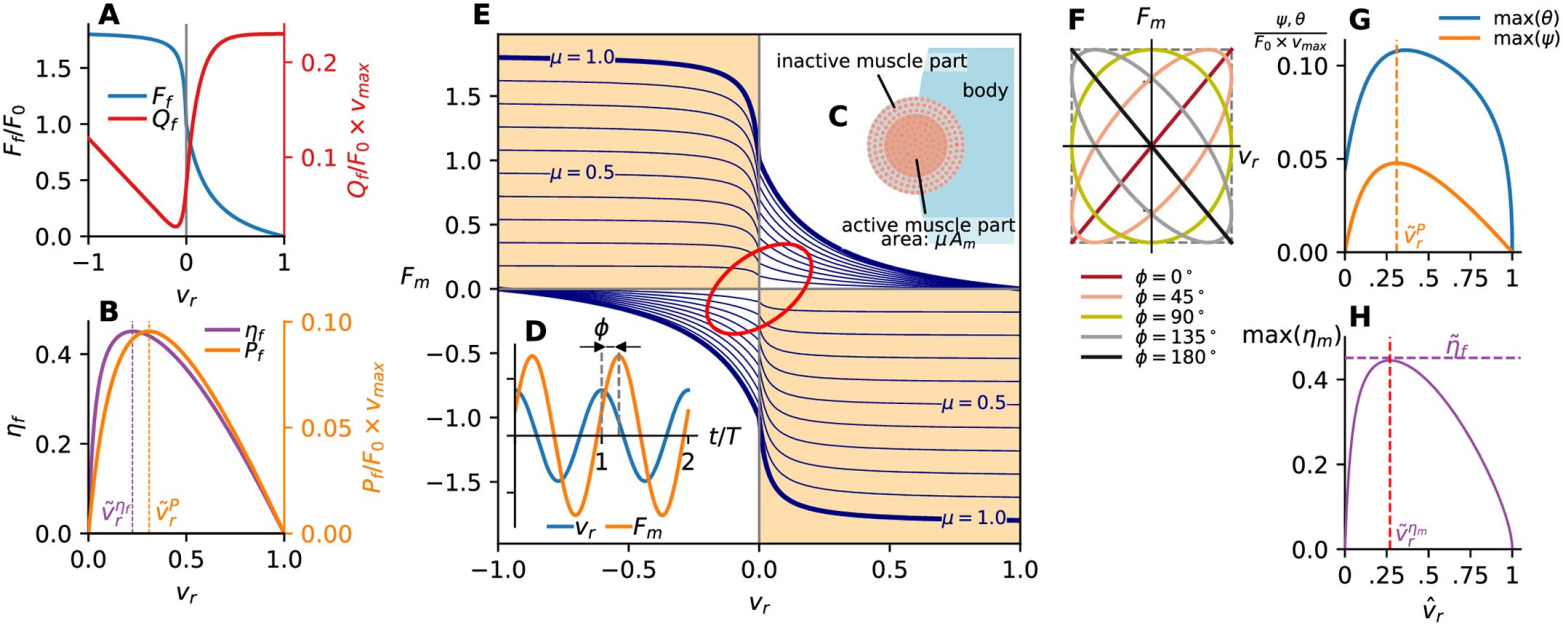
Muscle dynamics. (A) Hill’s model for the muscle fiber contraction force *F*_*f*_ and the associated power consumption *Q*_*f*_ as a function of the relative contraction speed *v*_*r*_ of the fiber. (B) Mechanical power production *p*_*f*_ and muscle fiber efficiency *η*_*f*_. (C) Sketch of the active part of the red muscle at a certain time instant. (D) Relative phase lag Φ between the periodic muscle force *F*_*m*_ and the periodic contraction speed *v*_*r*_. (E) A visualization of the muscle model for cyclic contraction performance in the cross section contracting with ${\hat{F}}_{m}=0.35$, ${\hat{v}}_{r}=0.20$, Φ = 60°. The force ellipse is marked in red. Maximum muscle force (full muscle used, i.e. muscle activation fraction *μ* = 1) is represented by thick blue lines; thin blue lines for *μ* < 1 isolines. The regions where muscle produces negative work (“braking”) is marked in orange. (F) Force ellipse shape as a function of phase lag Φ. (G) Maximum obtainable average powers over a cycle max(ψ) and max(θ) as a function of relative velocity amplitude ${\hat{v}}_{r}$ (Φ = 0). The orange dashed vertical line represents the values of ${\hat{v}}_{r}$ for which the average power output obtains an absolute maximum. (H) Maximum muscle efficiency max(*η*_*m*_) (Φ = 0) as a function of relative velocity amplitude ${\hat{v}}_{r}$. The red dashed vertical line marks the relative velocity amplitude ${\hat{v}}_{r}^{\eta_{m}}$ at which max(*η*_*m*_) is maximum.

We approach the muscle activation problem from an inverse direction: for a given undulatory body motion (of the form (1)), we calculate the required force the muscles have to produce (from a realistic force-velocity relationship) to sustain the motion and determine the muscle activation and performance based on it. The muscle model in our framework, illustratively summarized in Fig 2 (Fig 2E in particular), serves two main purposes: (I) to determine the necessary muscle force to produce the required bending moment *M*(*x*, *t*) for a feasible motion *h*(*x*, *t*), and (II) to determine the power produced by the muscles for such motion and the consequent metabolic power consumption. We consider motion powered solely by the superficial (red) muscle, Fig 1A and 1B, and assume that the muscle is longitudinally and laterally sufficiently innervated to allow different spatial and temporal muscle employment patterns [4].

#### Underlying assumption

The basis of our muscle model is the assumption that the force in a muscle fiber during steady swimming is a function of the instantaneous contraction velocity *v*(*x*, *t*) alone. During steady swimming, muscle fibers regularly operate on the plateau of length-tension curve [16]; thus, the effect of the fiber excursion on the contraction force can be neglected. Similarly, the characteristic time for muscle fibers to adapt to a new load is typically much shorter than the characteristic tail-beat period *T* of steady swimming [14, 33]. Hence, we assume quasi-steady muscle behavior, i.e. the muscle fibers contract with a constant velocity in tetanic contraction at every instant during the cycle.

For contraction with constant velocities *v*, the muscle fiber contraction force *F*_*f*_(*v*) and consumed power *Q*_*f*_(*v*) for the concentric (*v* > 0) and the eccentric contraction (*v* < 0) are given by the Hill’s model [33]. (The exact expressions are given in the Models section.) These are plotted in Fig 2A as a function of the relative contraction velocity *v*_*r*_ ≡ *v*/*v*_max_, where *v*_max_ is the maximum achievable contraction velocity for a particular fiber type; the contraction force is normalized by the isometric force *F*_0_ ≡ *F*_*f*_(0). The mechanical power output *P*_*f*_ = *F*_*f*_ × *v* produced by a fiber and the corresponding fiber efficiency *η*_*f*_ = *P*_*f*_/*Q*_*f*_ are plotted in Fig 2B. The fiber efficiency is defined only for *v*_*r*_ > 0, reaching the maximum of ${\tilde{\eta }}_{f}\equiv \max\eta_{f}=45.1\%$ at ${\tilde{v}}_{r}^{\eta_{f}}=0.23$. The maximum power output occurs at a slightly larger contraction velocity ${\tilde{v}}_{r}^{P}=0.31$. This forms a constitutive model that we use to build a model for the cyclic behavior of the entire muscle during swimming.

#### I. Motion and force feasibility

In order for the body motion *h*(*x*, *t*) to be physiologically feasible at every location along the body and at every instant, the following conditions have to be satisfied: (i) the fiber contraction speed *v*(*x*, *t*) has to be always smaller than *v*_max_, and (ii) the muscle cross-section area *A*_*m*_(*x*) has to be large enough to produce the required bending moment *M*(*x*, *t*) (given by Eq (9)) at every length-wise position.

The required contractive force *F*_*m*_(*x*, *t*) that muscles have to provide at any cross-section is *F*_*m*_(*x*, *t*) = *M*(*x*, *t*)/0.5 *b*(*x*), where 0.5 *b*(*x*) is the distance of the muscle cross-section from the body symmetry line. The sign of *F*_*m*_ uniquely determines the side of the body on which the muscles are active. By our convention, *F*_*m*_ is positive(negative) when the muscles on the right(left) side of the body are active. This, in turn, uniquely determines the sign of *v* in (2). In the case of linear underlying hydro-structural models (as we assume here, see Models), *F*_*m*_(*x*, *t*) is described by the same number of time harmonics as *h*(*x*, *t*). For *h*(*x*, *t*) given by (1), both *v*(*x*, *t*) and *F*_*m*_(*x*, *t*) contain only one time harmonic, i.e. they are purely sinusoidal in time.

At any cross section, the required muscle force *F*_*m*_ is the sum of all the active single-fiber contractive forces *F*_*f*_. To obtain the required force *F*_*m*_(*x*, *t*) constrained by *F*_*f*_(*v*(*x*, *t*)), we assume that only a fraction *μ*(*x*, *t*) of the total muscle cross-section area *A*_*m*_(*x*) is activated (Fig 2C):

$$\begin{array}{c} \mu \left(x,t\right)=\frac{|F_{m}\left(x,t\right)|}{F_{f}\left(v\left(x,t\right)\right)\;A_{m}\left(x\right)}\;. \end{array}$$ (3)

This expression for the active muscle fraction *μ*(*x*, *t*) determines both the physiological feasibility of motion (the requirement that *μ* ≤ 1) and the way how the muscle force *F*_*m*_ is provided (by activating *μA*_*m*_ of the muscle cross section).

#### II. Resulting energetic quantities

The instantaneous metabolic power *q*(*x*, *t*) consumed by the muscle fibers at any cross section is the sum of the power consumed by all active muscle fibers at that instant and location, resulting in *q*(*x*, *t*) = *μ*(*x*, *t*)*A*_*m*_(*x*)*Q*_*f*_(*v*(*x*, *t*)). The power consumption is always positive, reflecting the physical fact that muscles always consume metabolic energy. At the same time, the muscles at every cross section produce mechanical power output *p*(*x*, *t*) = *F*_*m*_(*x*, *t*)*v*(*x*, *t*). It can be positive or negative, i.e. the muscles are either providing useful work or extracting energy from the system by contracting while being extended by external forces (eccentric contraction). As both *F*_*m*_ and *v* are sinusoidal, the indicator of the overall character of the mechanical power output at some cross section is the relative phase difference Φ(*x*) between these two quantities, Fig 2D. For 0 ≤ Φ < 90°, the mechanical power output *p* is mostly positive during the motion cycle ($\bar{p}>0$, where $\bar{\left(\cdot \right)}$ denotes the time average) and the muscles are in the “power” mode; for 90° < Φ(*x*) ≤ 180°, *p* is mostly negative ($\bar{p}<0$). To simplify the terminology, we will refer to the regime where negative net work is being done, i.e. the muscles are predominantly in eccentric contraction, as the “braking” mode. For Φ = 0, the mechanical power is positive during the entire cycle; the opposite is true for Φ = 180°. For Φ = 90°, the average mechanical power output $\bar{p}$ is zero. The average metabolic power consumption $\bar{q}$ in all these cases is positive.

The maximum values average power consumed or produced over a cycle occur for Φ = 0, so they depend on the relative contraction velocity amplitude ${\hat{v}}_{r}$ alone. The maximum values of the average power production and consumption per muscle area $\psi \equiv \bar{p}/A_{m}$ and $\theta \equiv \bar{q}/A_{m}$ are shown in Fig 2G. As a consequence of the cyclic (sinusoidal) muscle operation, the maximum average power output per muscle area max(*ψ*) is exactly one half of the maximum power output for fibers contracting with a constant velocity, and it occurs at the same relative contraction velocity ${\tilde{v}}_{r}^{P}=0.31$. For characteristic muscle properties used in this work (see Models), the maximum obtainable power output per muscle mass based on our model is $\tilde{\psi }=35.8\;\text{W}/\text{kg}$, a value comparable to those found in nature [7, 15]. Away from ${\tilde{v}}_{r}^{P}$, max(*ψ*) is monotonically decreasing. The maximum values of average consumed power per muscle area *θ* as a function of ${\hat{v}}_{r}$ exhibit an overall maximum at ${\hat{v}}_{r}$ very close to ${\tilde{v}}_{r}^{P}$. For the chosen muscle properties, the maximum power consumption per muscle mass is $\tilde{\theta }=81.2\;\text{W}/\text{kg}$. Unlike max(*ψ*), max(*θ*) is nonzero at ${\hat{v}}_{r}=0$ (for a fully activated muscle, max(*θ*)(0) = 2/*π* × *Q*_*f*_(0)).

The efficiency of producing mechanical work at a particular location can be quantified by the local muscle efficiency *η*_*m*_. At any cross-section, we define *η*_*m*_ as the ratio between the average mechanical power produced over a cycle and the average metabolic power consumed over that cycle, i.e.

$$\begin{array}{c} \eta_{m}\left(x\right)\equiv \frac{\bar{p}\left(x\right)}{\bar{q}\left(x\right)}\;. \end{array}$$ (4)

The local muscle efficiency is meaningfully defined only for the cross sections where $\bar{p}\left(x\right)$ is positive, i.e. muscles are producing positive net power (phase lag Φ < 90°). The maximum values of *η*_*m*_ are attained for Φ = 0 and are a function of ${\hat{v}}_{r}\left(x\right)$ only, Fig 2H. The overall maximum of *η*_*m*_ based on our model is ${\tilde{\eta }}_{m}=44.5\%$ and it occurs when the amplitude of relative contraction velocity is ${\tilde{v}}_{r}^{\eta_{m}}=0.27$; *η*_*m*_ monotonically decreases away from ${\tilde{v}}_{r}^{\eta_{m}}$. Note that ${\tilde{\eta }}_{m}$, which represents the maximum efficiency of cyclical contractions of muscle fibers, is only marginally smaller than the maximum fiber efficiency ${\tilde{\eta }}_{f}$, which measures the fiber efficiency when contracting with constant *v*_*r*_. It is also comparable to the empirically obtained maximum efficiency of red muscle fibers of 51% [34].

#### Muscle diagram

The dependency between the contraction velocity, the required muscle force, the maximum available muscle force, and the mode in which the muscle is operating at a certain cross section *x*_0_ can be illustrated by a four-quadrant force-velocity diagram, Fig 2E. Here, the muscle force *F*_*m*_(*x*_0_, *t*) is plotted as a function of contraction velocity *v*_*r*_(*x*_0_, *t*) such that the positive(negative) values of *F*_*m*_ correspond to the activation of the fibers on the right(left) side of the body, (respectively). The maximum force the muscle can produce *F*_*f*_(*v*_*r*_) × *A*_*m*_(*x*_0_), corresponding to the full muscle activation *μ* = 1, provides a limit that *F*_*m*_ cannot exceed. Note that for the left side muscle activation, the *μ* = 1 envelope is plotted proportional to −*F*_*f*_(−*v*_*r*_) to account for our sign convention.

In the *F*_*m*_–*v*_*r*_ coordinate system, the contraction velocity *v*_*r*_ and the muscle force *F*_*m*_ form what we term a *force loop*, which is an ellipse in the case of a sinusoidal *F*_*m*_ and *v*_*r*_. Time is only implicitly (parametrically) present in the force ellipse. A physiologically feasible motion is any motion for which the force ellipse is entirely contained within the *μ* = 1 lines. The activation fraction *μ*(*x*_0_, *t*) for a certain point on the force ellipse (i.e. *F*_*m*_(*x*_0_, *t*)–*v*_*r*_(*x*_0_, *t*) pair) is determined from the intersection of the ellipse with a particular *μ*-isoline, which is a proportional reduction of the *μ* = 1 line. The amplitudes ${\hat{F}}_{m}$ and ${\hat{v}}_{r}$ of *F*_*m*_ and *v*_*r*_ determine the bounding box of the force ellipse, and the ellipse shape and the orientation of its major axes depend on the phase difference Φ, Fig 2F. For Φ = 0° and Φ = 180° (pure power and braking modes), the ellipse reduces to a line.

#### Time response

**Fig 3:**
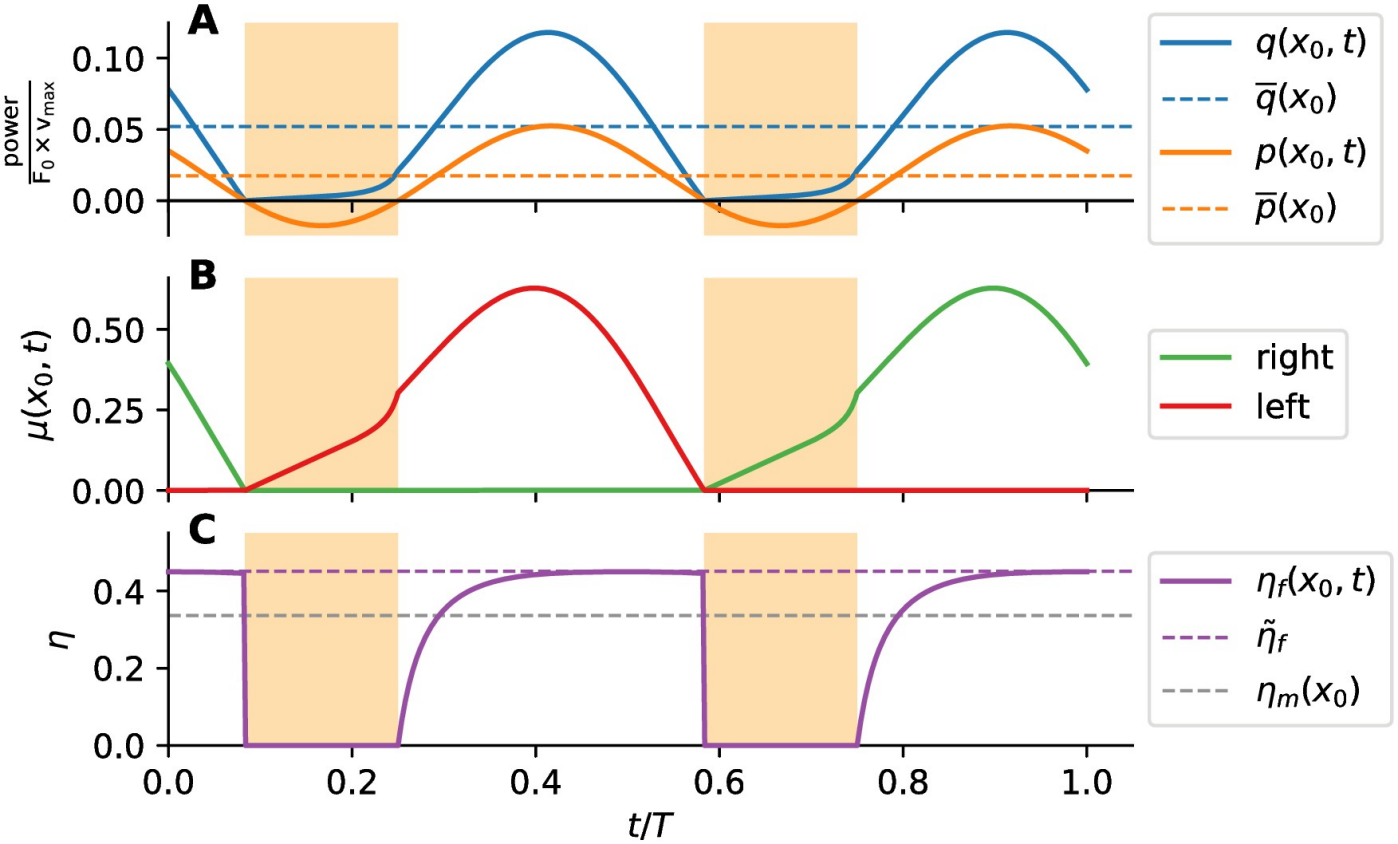
Time response of a muscle in oscillatory contraction. The plots represent the time response at a cross section *x*_0_ where the muscle is contracting with ${\hat{F}}_{m}=0.35$, ${\hat{v}}_{r}=0.20$, Φ = 60°. Intervals of eccentric contraction (“braking”) are marked in orange. Time *t* is normalized by the tail-beat period *T*. (A) Metabolic power consumption *q* (blue line) and mechanical power production *p* (orange line). The average values $\bar{p}$ and $\bar{q}$ are represented by dashed lines in corresponding color. (B) Muscle activation fraction *μ*(*x*_0_, *t*). Green line represents the active fraction of the muscle on the right side, red line for that on the left side. (C) Muscle fiber efficiency *η*_*f*_. The resulting local muscle efficiency *η*_*m*_(*x*_0_) is shown in dashed gray line. Maximum fiber efficiency ${\tilde{\eta }}_{f}$ from Hill’s model is shown in dashed magenta line.

The time dependency of the relevant quantities, which is only implicitly present in Fig 2E, is explicitly shown in Fig 3 for a certain cross section *x*_0_. The intervals during which the muscle fibers at *x*_0_ are producing negative work *p*(*x*_0_, *t*) < 0, i.e. braking, are marked in all plots. Since Φ(*x*_0_) < 90° for this cross section, a smaller fraction of the tail-beat period is spent in the braking mode. The instantaneous consumed power *q*(*x*_0_, *t*) is always positive and greater than *p*(*x*_0_, *t*). The intermittent character of the muscle fiber activation is shown in Fig 3B. The fibers on only one side are active at any given time. The muscle fibers on each side undergo the concentric and eccentric contraction (braking) intervals. The kink in *μ*(*x*_0_, *t*) at the end of every braking interval is due to the kink in *F*_*f*_ at *v*_*r*_ = 0 (cf. Fig 2E). The efficiency of active fibers *η*_*f*_ as a function of time in shown in Fig 3C. Note that *η*_*f*_ is not defined during the braking intervals. Outside the braking intervals, *η*_*f*_ is close to the maximum achievable ${\tilde{\eta }}_{f}$ for a significant portion of the cycle. The local muscle efficiency *η*_*m*_(*x*_0_), which accounts for the time averaged power transfer, is greater than zero (*η*_*m*_(*x*_0_) = 33.6% here) since Φ < 90°, but still lower than ${\tilde{\eta }}_{f}$. For a cross section where Φ > 90°, the average power output would be negative, so *η*_*m*_ for that cross section would be zero. To account for the contributions from all cross sections, we consider length-integrated time-averaged energetic measures next.

### Integral energetic measures

Arguably the most important energetic measure of swimming (and locomotion in general) is the cost of transport (COT), a non-dimensional measure that accounts for the absolute amounts of consumed energy. COT is defined as the total energy spent per unit mass and distance traveled [4, 35], which can be expressed as

$$\begin{array}{c} \text{COT}\equiv \frac{P_{T}}{mgU}=\frac{P_{s}+Q}{mgU} \end{array}$$ (5)

where *P*_*T*_, the total metabolic power consumed while swimming at the speed *U*, is the sum of total the metabolic power *Q* consumed for swimming (length-integrated, time-averaged *q*(*x*, *t*)), and the speed-independent standard metabolic rate *P*_*s*_ required for other physiological processes; *g* is the acceleration of gravity and is used only for nondimensionalization purposes here. As such, COT is a “gallons-per-mile” measure and is likely to be the governing criterion for long migrations [4]. Since COT is normalized by the body mass, it also serves as a comparative measure of energy consumption across the scales.

The process of energy conversion during swimming can be separated into three parts—the conversion of metabolic energy acquired by food into mechanical work through the contracting muscle fibers, the conversion of that mechanical work into the work passed to the fluid, and the conversion of the fluid energy into the propulsion work, the final useful energy form. The corresponding efficiencies of these conversion processes can be labeled as the muscle, the internal and the hydrodynamic efficiency, respectively.

We define the muscle efficiency *η*_*M*_ as the efficiency in converting the total consumed metabolic energy into mechanical work

$$\begin{array}{c} \eta_{M}\equiv P/Q\;, \end{array}$$ (6)

where *P* is the total mechanical work produced by the muscles per tail-beat cycle, i.e length-integrated, time-averaged power output. The total produced work is always positive since the muscles power swimming. Therefore, *η*_*M*_ is always meaningfully defined. The muscle efficiency *η*_*M*_ is bounded from above by the local muscle efficiency *η*_*m*_, setting the upper limit of *η*_*M*_ to $\max\eta_{M}=\max\eta_{m}\left(x\right)={\tilde{\eta }}_{m}=44.5\%$ as well.

The work produced by the muscles to power the swimming motion is only partly transferred to the fluid because some of it is used to overcome the internal visco-elastic losses occurring in the tissues. We quantify these losses through the internal efficiency *η*_*I*_, which relates the power transferred to the surrounding flow and the work produced by the muscles. The internal efficiency could also be combined with the muscle efficiency to produce a combined “muscle” efficiency $\eta_{M}^{*}=\eta_{M}\;\eta_{I}$ [3]. For real organisms with non-zero visco-elastic losses, $\eta_{M}^{*}<\eta_{M}$. The use of *η*_*M*_ and *η*_*I*_ allows for a more detailed insight into the energy conversion process by accounting for where the energy losses are.

The hydrodynamic (Froude) efficiency *η*_*H*_ is commonly defined as the ratio between the useful power for propulsion $\bar{F_{T}}U$ (where $\bar{F_{T}}$ is the time-averaged thrust force) and the average power transferred to the surrounding fluid by the body motion [31]. Taking the entire energy conversion process into account, the total swimming efficiency *η*_*T*_ can be written as

$$\begin{array}{c} \eta_{T}\equiv \frac{\bar{F_{T}}U}{Q}=\eta_{H}\;\eta_{M}\;\eta_{I}\;. \end{array}$$ (7)

The efficiencies, unlike COT, are ratios of average powers and as such cannot differentiate between the absolute amounts when the ratios are the same. This is a major drawback of using efficiencies as energetic measures for optimization in cases when neither the produced nor the consumed power is prescribed, as it is the case in our optimization problem.

### Optimal populations

In this study, we focus on the multi-objective optimization of swimming organisms with respect to two conflicting performance measures of arguably great importance in the evolutionary scenario [2, 4]: maximizing the sustained swimming speed *U* and the minimizing cost of transport COT. Simultaneously optimizing conflicting objectives usually leads to an infinite number of optimal solutions. In the multi-objective context, a solution is considered optimal when it is non-dominated, i.e. when there is no feasible variation of optimization variables that could improve every objective. We call the set of non-dominated organisms the optimal population Π.

**Fig 4:**
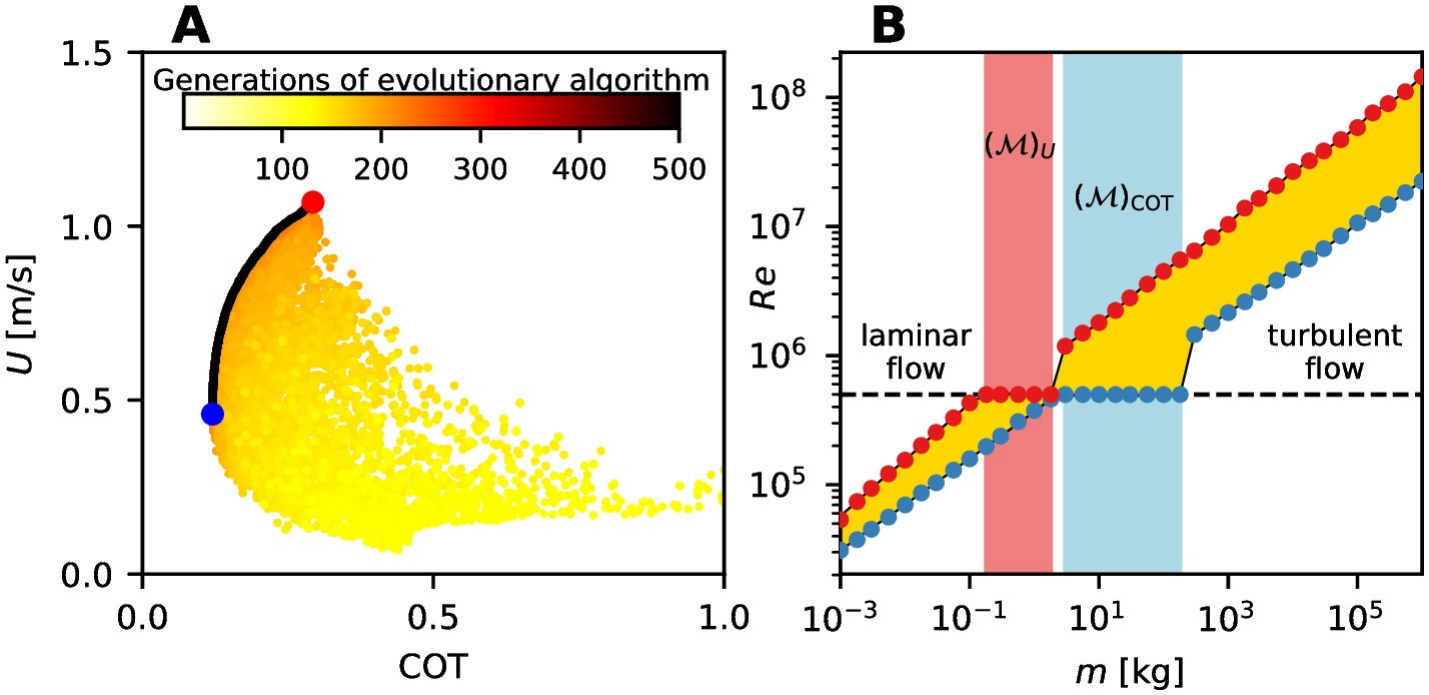
Optimal populations and Pareto front. (A) Pareto front (black) representing the optimal (non-dominated) population Π(*m*); non-optimal (dominated) solutions behind the Pareto front are also shown, colored according to the generation of the evolutionary algorithm they belong to. No solution among the dominated ones is better in both objectives (i.e. higher *U* and lower COT) than the solutions on the Pareto front. The COT-optimal solution is marked by a blue dot, the *U*-optimal by a red dot. (B) Reynolds number *Re* of optimal organisms (from [30]). Optimal populations are represented by the yellow area. Red dots correspond to *U*-optimal organisms, blue dots to COT-optimal ones. The transition ranges ${\left(M\right)}_{U}$ and ${\left(M\right)}_{\text{COT}}$ are marked in red and blue regions, respectively.

In the two-dimensional objective space, the non-dominated solutions Π form a so-called Pareto front, which illustrates the functional trade-offs between the conflicting objectives, e.g. Fig 4A. In this space, solutions that optimize a single objective alone are always at the ends of the Pareto front. In a general space, these single-objective optimal organisms do not necessarily obtain extreme values within the optimal population. In our results, we focus particularly on the representatives from the optimal populations for which either *U* or COT is optimal. The values corresponding to those solutions are henceforth denoted by (⋅)_*U*_ or (⋅)_COT_, respectively.

For any quantity *χ* characterizing swimming performance across many scales in body mass *m*, the values of *χ* that optimal populations Π(*m*) obtain form a band when plotted as a function of *m*; for example Fig 4B for *χ* ≡ *Re*. When we observe (*χ*)_*U*_ or (*χ*)_COT_ values across the scales in *m*, the behavior of swimmers of intermediate sizes (among those that we consider) is noticeably different from that of either smaller organisms that swim in a laminar regime or larger organisms that swim in a turbulent one. This common change in scaling behavior occurs when organisms of increasing *m* abandon the *Re*–*m* scaling at some value of *m* (corresponding to the critical Reynolds number *Re*_*cr*_) to remain in the laminar regime, Fig 4B. We call the range of *m* over which this occurs a transition range M. The optimal swimmers in M maintain the subcritical Reynolds number by being of shorter length and/or by swimming at a lower speed, relative to the scaling observed for the range of *m* below M. The width of M (in terms of the range of *m*) and the value of *m* at which M begins is generally different for *U*-optimal and COT-optimal organisms. Given that *U*-optimal organisms are faster than COT-optimal ones for any *m*, the transition range ${\left(M\right)}_{U}$ for the *U*-optimal organisms starts and ends for lower *m* than the ${\left(M\right)}_{\text{COT}}$ one.

## Results

We present here the energy-related quantities of optimal swimming organisms, obtained from a multi-objective optimization of swimmers covering a large range of body sizes (from *m* = 0.001 kg to *m* = 1, 000, 000 kg). The swimmers are simultaneously optimized with respect to maximizing the sustained swimming speed *U* and minimizing the cost of transport COT.

The optimization of nine variables parameterizing the body shape and motion was performed using the MO-CMA-ES evolutionary algorithm [36], starting from initial populations with random shape and motion parameters (for more details, see Models). Note that apart from the body wavelength, no kinematic or dynamic quantity is prescribed, i.e. all the quantities are outcomes of the optimization process. The assumed body and muscle properties are realistic and considered constant for all swimmers (see Models section). The total number of simulation evaluations required to obtain the converged results for all body sizes presented here is *O*(10^7^).

We present the results of relevant integrated and distributed internal energetic quantities, and show that they are consistent and in agreement with the empirically obtained ones across the scales. These results complement our previously published results on the shapes, motion patterns and kinematic quantities of optimal populations in this size range [30]. Note that some of the results are quantitatively dependent on the values of the assumed muscle properties (*F*_0_, *v*_max_ and *μ*_0_).

### Optimal COT is inversely proportional to maximum efficiency

**Fig 5:**
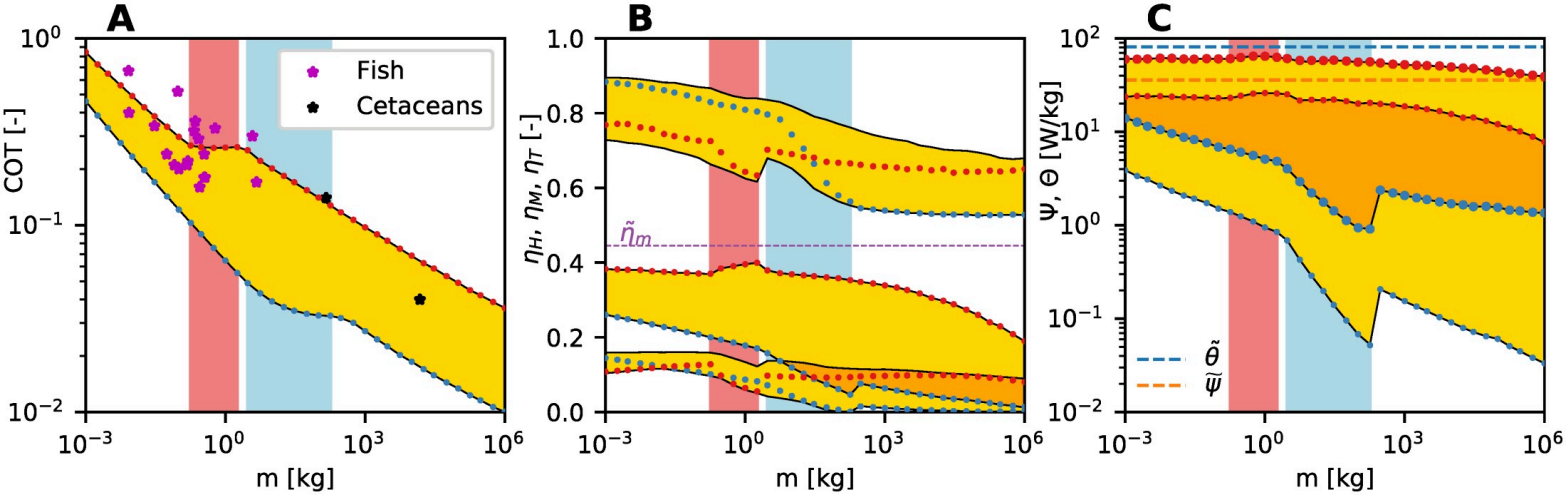
Energetic characteristics of optimal populations Π(*m*) across the scales. Organisms with minimum COT are marked by blue circles and *U*-optimal organisms by red circles; the rest of Π(*m*) are represented by yellow region(s). The transition range ${\left(M\right)}_{U}$ is marked by a red region and ${\left(M\right)}_{\text{COT}}$ by a blue region. (A) Cost of transport COT. Empirical data [4] for fish (magenta stars) and cetaceans (bottlenose dolphin and gray whale; black stars) are shown for comparison. (B) Hydrodynamic efficiency *η*_*H*_ (upper region), muscle efficiency *η*_*M*_ (middle region) and total efficiency *η*_*T*_ (lower region). The dashed magenta line represents the maximum achievable muscle efficiency ${\tilde{\eta }}_{m}$ of 44.5%. (C) Mechanical power production per muscle mass Ψ (lower region, small circles) and metabolic power consumption per muscle mass Θ (upper region, large circles). The dashed horizontal lines represent the maximum obtainable power per muscle mass $\tilde{\psi }$ and $\tilde{\theta }$ for the selected muscle properties. The intersection of optimal regions in B and C is shown in a darker shade of yellow.

The cost of transport, power production/consumption and accompanying efficiencies of optimal populations Π(*m*) across the scales of body mass *m* are shown in Fig 5. For every *m*, these quantities obtain a range of values, each corresponding to one of the non-dominated solutions from Π(*m*).

The cost of transport COT of optimal swimmers continuously decreases with *m* (except in the transition ranges M), Fig 5A. The rate of the decrease matches that of fish and cetaceans [4]. The COT-optimal and *U*-optimal organisms achieve extreme values of COT within each optimal population Π(*m*), being at a minimum for (COT)_COT_ and at a maximum for (COT)_*U*_. This is expected due to the conflicting nature of the two optimization objectives. These COT values were already reported in [30]; we present them here for completeness and to contrast them with the other energetic values.

Unlike COT, the total swimming efficiency *η*_*T*_ does not exhibit a consistently minimum or maximum values for either *U*-optimal or COT-optimal organisms, Fig 5B. Surprisingly, swimming at minimum COT is not correlated with the maximum efficiency, i.e. with what one might consider as energy-efficient swimming, but rather to the *minimum* of efficiency. The key to this behavior lies within the muscle efficiency *η*_*M*_. The COT-optimal organism consistently achieve minimum *η*_*M*_ within the optimal populations Π(*m*). Furthermore, (*η*_*M*_)_COT_ is decreasing with an increase in body size. Having a minimum *η*_*M*_ within the optimal population is obviously no impediment to having the optimal cost of transport (cf. Fig 5A). In contrast, (*η*_*M*_)_*U*_ is constantly maximum within Π(*m*), reaching the values close to maximum attainable ${\tilde{\eta }}_{m}=44.5\%$ for body sizes at the upper end of ${\left(M\right)}_{U}$.

The hydrodynamic efficiency *η*_*H*_ of optimal populations Π(*m*) reaches values between 55%–90%. The values of (*η*_*H*_)_*U*_ occur within a narrow range, roughly between 65%–75%. In contrast, (*η*_*H*_)_COT_ covers a greater range; it is higher than (*η*_*H*_)_*U*_ for smaller body sizes and falls below it for body sizes beyond the transition range ${\left(M\right)}_{\text{COT}}$. The hydrodynamic efficiency is never maximum or minimum for COT-optimal and *U*-optimal organisms (except for (*η*_*H*_)_COT_ for large *m*); the extreme values of *η*_*H*_ are generally achieved by some other members of the optimal population Π(*m*). The overall values of *η*_*H*_, however, might be slightly over-predicted by the simple hydrodynamic model that we use in the present study, as has been indicated by some CFD studies [21].

The predicted muscle efficiency *η*_*M*_ of optimal populations for smaller body sizes is roughly in the range of 20%–40%, but for larger body sizes, *η*_*M*_ generally decreases to values as low as 2%–20%. The corresponding values of $\eta_{M}^{*}$ are in the 1.5%–20% range. Thus, the total efficiency *η*_*T*_, despite relatively high *η*_*H*_, is in the ∼2%–16% range. The range of values for $\eta_{M}^{*}$ and *η*_*T*_ are in agreement with previously reported values for fish [3].

### Power scales significantly different for *U*-optimal and COT-optimal organisms

To asses the energetic utilization of the available muscle in optimal swimmers across the scales, we study the total power production *P* and consumption *Q* per muscle mass *m*_*M*_. For notational convenience, we define Ψ ≡ *P*/*m*_*M*_ and Θ ≡ *Q*/*m*_*M*_ as power produced and consumed per muscle mass measures, respectively.

The values of Ψ and Θ scale significantly different for *U*-optimal and COT-optimal organisms, Fig 5C. The maximum values within Π(*m*) are consistently achieved for *U*-optimal swimmers, and the obtained (Ψ)_*U*_ and (Θ)_*U*_ values are almost constant across the scales of body sizes (even through the transition range ${\left(M\right)}_{U}$), decreasing only slightly for very large *m*. The maximum power-per-mass values for *U*-optimal swimmers are not surprising since it can be expected that organisms at every size utilize all the available muscle fibers to achieve maximum sustained swimming speeds [15]. On the other hand, the powers obtained for COT-optimal organisms (Ψ)_COT_ and (Θ)_COT_ consistently acquire minimum values among Π(*m*). As a consequence of *η*_*M*_-scaling, (Ψ)_COT_ and (Θ)_COT_ values exhibit a significant decrease with an increase in *m*; the rate is faster for (Ψ)_COT_. Within the transition range ${\left(M\right)}_{\text{COT}}$, the decrease rate is even more pronounced.

The range of Ψ values obtained from our calculations is largely within the reported empirical values. The measurements of the muscle fiber power production of swimming fish that are up to 10 kg in body size give the values of roughly between 1 W/kg–30 W/kg (skipjack tuna being an outlier at 100 W/kg) [15], which covers the range predicted here.

### Longitudinal distribution of energetic quantities depends on the optimization objective

The single-valued energetic measures presented in Fig 5 give only a part of the picture of the swimming energetics of optimal organisms. Since energy is being consumed and useful work produced throughout the body, we present next the longitudinal distributions of relevant quantities to gain a deeper insight into the workings of the locomotory muscles.

**Fig 6:**
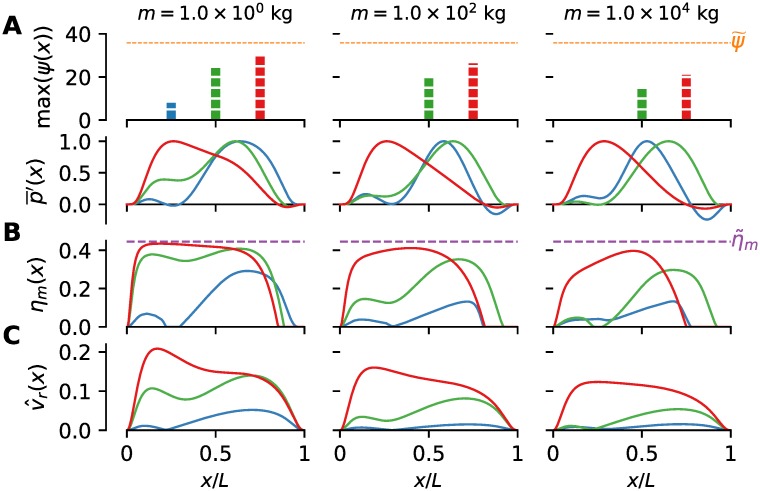
Longitudinal distributions of energetic quantities across the scales. Different representatives from optimal populations are denoted by colors: (⋅)_COT_ quantities in blue, (⋅)_*U*_ in red, and quantities related to the intermediate representative from the Pareto front in green. (A) Normalized average mechanical power ${\bar{p}}^{\prime }\left(x\right)$. For clarity, all quantities have been normalized with their corresponding maximum values due to a large difference in scales. These maximum values for each swimmer are shown in the bar plot above, expressed as power per muscle mass *ψ*, in corresponding color for each representative. The maximum obtainable power output per muscle mass $\tilde{\psi }=35.8\;\text{W}/\text{kg}$ is shown in dashed orange line. (B) Local muscle efficiency *η*_*m*_(*x*). The dashed magenta line marks the theoretically maximum achievable muscle efficiency (${\tilde{\eta }}_{m}=44.5\%$). (C) The envelope ${\hat{v}}_{r}\left(x\right)$ of the relative contraction velocity.

We have chosen three representatives from Π(*m*) at three characteristic body sizes to show how the envelope of the relative contraction velocity ${\hat{v}}_{r}\left(x\right)$, the local muscle efficiency *η*_*m*_(*x*) and the power production/consumption vary along the length, Fig 6. Two representatives correspond to the extremes of the Pareto front, i.e. *U*-optimal and COT-optimal representatives, while the third representative is chosen from the middle of the Pareto front. The characteristics of other optimal organisms can generally be smoothly interpolated between the presented characteristics, both along the Pareto front and across the scales. In the following discussion, we will focus on the *U*-optimal and COT-optimal characteristics, as the characteristics for the third representative are usually in between these two.

The distribution of the average mechanical power output $\bar{p}\left(x\right)$ of optimal populations show consistent characteristics across the scales, Fig 6A. The anterior part of *U*-optimal swimmers produces most of the power, as indicated by the location of maxima of ${\left(\bar{p}\left(x\right)\right)}_{U}$. Compared to the posterior, the anterior part of the *U*-optimal swimmers becomes even more energetically dominant with an increase in body size. Note that the decrease in total power levels in the caudal region is in part due to the reduced muscle mass, proportionally to the reduction in the cross-section area. For COT-optimal organisms, the power is mostly produced mid-body, or slightly aft. With an increase in body size, there is a notable growth of the braking region in the caudal area where the the power production ${\left(\bar{p}\left(x\right)\right)}_{\text{COT}}$ is negative.

The local efficiency (*η*_*m*_(*x*))_*U*_ is almost always consistently greater than (*η*_*m*_(*x*))_COT_, reaching its maximum achievable value of 44.5% for most of the anterior part of the body for smaller body sizes. A prominent feature of (*η*_*m*_(*x*))_COT_ for all but the largest body sizes is the existence of two local maxima of *η*_*m*_, separated by (nearly) zero efficiency in the middle. The maximum of (*η*_*m*_(*x*))_COT_ is generally located in the posterior half of the body. Note that (*η*_*m*_(*x*))_*U*_ and (*η*_*m*_(*x*))_COT_ are generally not defined in the tail region where $\bar{p}$ becomes negative (“braking” mode). With an increase in body size, the “braking” region enlarges.

The maximum longitudinal value of the envelope ${\hat{v}}_{r}\left(x\right)$ of the relative contraction velocity *v*_*r*_(*x*, *t*) is always less than ${\tilde{v}}_{r}^{\eta_{m}}=0.27$ for which *η*_*m*_ is maximum, and it is generally decreasing with an increase in body size, Fig 6C. The decrease in the maximum values of ${\hat{v}}_{r}$ with *m* is due to the fact that the tail-beat frequency *ω* is also decreasing with an increase in *m*, while the maximum achievable contraction velocity *v*_*max*_ is constant. The values of ${\left({\hat{v}}_{r}\left(x\right)\right)}_{U}$ are always greater than ${\left({\hat{v}}_{r}\left(x\right)\right)}_{\text{COT}}$, indicating that faster contraction rates correspond to faster swimming, which is intuitive. For ${\hat{v}}_{r}<{\tilde{v}}_{r}^{\eta_{m}}$, ${\hat{v}}_{r}$ is positively correlated with *η*_*m*_(*x*) and $\bar{p}$ so that a relatively higher ${\hat{v}}_{r}\left(x\right)$ corresponds to higher efficiency *η*_*m*_(*x*) and higher power output $\bar{p}$.

Larger values of ${\left({\hat{v}}_{r}\left(x\right)\right)}_{U}$ are found anteriorly, especially for smaller organisms where ${\left({\hat{v}}_{r}\left(x\right)\right)}_{U}$ is close to ${\tilde{v}}_{r}^{\eta_{m}}$, producing the regions of nearly maximum *η*_*m*_(*x*). The character of ${\left({\hat{v}}_{r}\left(x\right)\right)}_{\text{COT}}$ is similar to that of (*η*_*m*_(*x*))_COT_ in that it exhibits two regions with higher values, with (nearly) zero values of ${\hat{v}}_{r}\left(x\right)$ in between. The locations of zero values of ${\left({\hat{v}}_{r}\left(x\right)\right)}_{\text{COT}}$ approximately correspond to the locations of the minima of motion envelopes (*r*(*x*))_COT_ [30].

**Fig 7:**
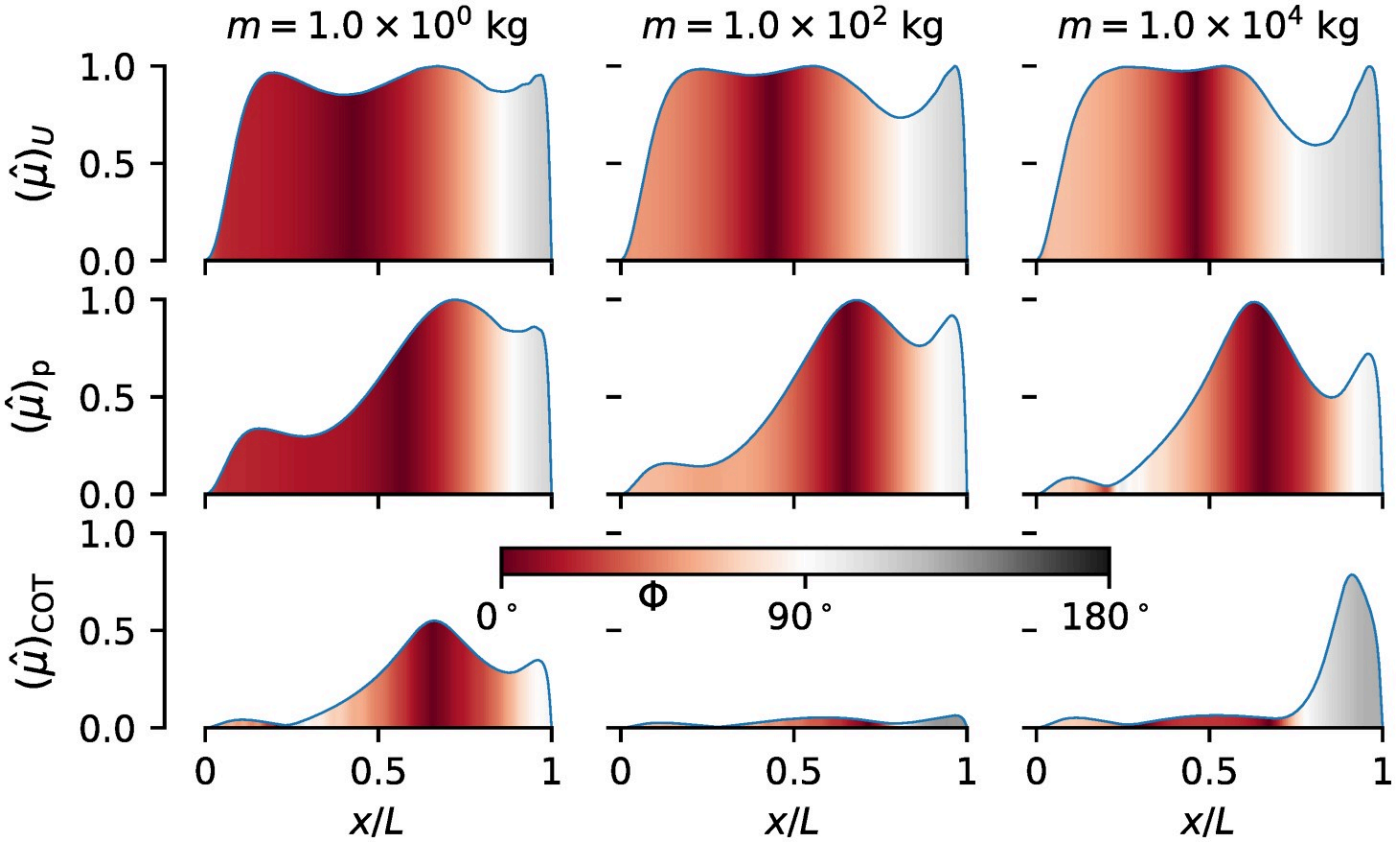
Muscle activation envelope across the scales for select optimal representatives from the Pareto front. The fill color represents the corresponding phase lag Φ(*x*). The top row corresponds to ${\left(\hat{\mu }\left(x\right)\right)}_{U}$, the bottom one to ${\left(\hat{\mu }\left(x\right)\right)}_{\text{COT}}$, while the middle row ${\left(\hat{\mu }\left(x\right)\right)}_{\text{p}}$ corresponds to optimal organisms from the middle of the Pareto front, like in Fig 6.

Consideration of the maximum muscle activation fraction along the length $\hat{\mu }\left(x\right)$ (the envelope of *μ*(*x*, *t*)) together with the longitudinal distribution of the phase lag Φ(*x*), Fig 7, provides additional insights into the effectiveness of muscle performance. The presented results correspond to the same optimal individuals as in Fig 6 and can be smoothly interpolated between those shown.

All *U*-optimal organisms achieve $\hat{\mu }=1$ at some point along the length, as the maximum achievable sustained speed is constrained by the available muscle. Usually, ${\left(\hat{\mu }\left(x\right)\right)}_{U}\approx 1$ for the majority of the body length. With an increase in body size, there is a notable decrease in the active muscle fraction ${\left(\hat{\mu }\left(x\right)\right)}_{U}$ in the caudal peduncle area. Nearly maximum ${\left(\hat{\mu }\left(x\right)\right)}_{U}$ offers part of the explanation for maximum values of (*P*)_*U*_ and (*Q*)_*U*_ within the optimal populations—as more muscle fibers are employed, the power levels are higher. The other part comes from the way the muscles are operating. The phase lag (Φ(*x*))_*U*_ is nearly 0° for the anterior part of the body, especially for smaller *m*, indicating large average power output. In the caudal region, however, (Φ(*x*))_*U*_ is generally greater than 90°, indicating that the caudal muscles are used for braking. These large values of Φ cause a decrease in $\bar{p}\left(x\right)$ (Fig 6A), despite the large muscle activation $\left(\hat{\mu }\left(x\right)\approx 1\right)$.

COT-optimal organisms are generally not constrained by the available muscle. The active muscle fraction ${\left(\hat{\mu }\left(x\right)\right)}_{\text{COT}}$ is larger in the posterior half of the body, but is generally less than 1. For smaller body sizes (below ${\left(M\right)}_{\text{COT}}$), the maximum value of ${\left(\hat{\mu }\left(x\right)\right)}_{\text{COT}}$ along the length decreases with an increase in body mass *m*, reaching its minimum for *m* around 100 kg. For larger body sizes (above ${\left(M\right)}_{\text{COT}}$), the maximum value of ${\left(\hat{\mu }\left(x\right)\right)}_{\text{COT}}$ increases with the increase in *m*. This behavior has already been reported [30]. For very large organisms, the increase in ${\left(\hat{\mu }\left(x\right)\right)}_{\text{COT}}$ is concentrated in the tail region, exhibiting an abrupt peak in the values. The phase lag (Φ(*x*))_COT_ exhibits similar behavior to (Φ(*x*))_*U*_, with muscles in the caudal region producing mostly negative work (braking). The anterior regions for small COT-optimal organisms, where muscles do almost no net work ((Φ(*x*))_COT_ ≈ 90°), are of little importance since $\hat{\mu }⪡1$ there.

## Discussion

The presented results, obtained by extensive computational simulations based on a coupled muscle-structural-hydrodynamic model, offer a comprehensive look into the undulatory swimming energetics, both within a swimming body and across the scales. Such a coupled analysis has not been reported in the literature, and we argue that without understanding the dynamics of both the muscle behavior and the surrounding flow, we cannot fully understand the dynamics of swimming and the evolutionary trade-offs that might have taken place in fish and cetaceans. To that end, we have provided a deeper analysis of our muscle model (first introduced in [30]) that is an integral part of our framework, and introduced the concept of a force loop (Fig 2). Our results provide further confirmation that the optimization of swimming organisms with respect to the maximization of the sustained swimming speed *U* and the minimization of the cost of transport COT—two conflicting, evolutionary important performance measures—leads to optimal populations that have energetic characteristics similar to those found in nature. The conflicting nature of the performance measures contributes to the diversity of obtained behavior.

We limit the discussion to sustained, continuous undulatory swimming alone. Some fish at certain swimming speeds employ intermittent propulsion, where a short burst of undulatory motion is periodically followed by a stretched-straight, no-power coasting phase [37]. There are indications that this burst-and-coast swimming could lead to energetic savings over the continuous undulatory swimming [38, 39]. However, since it is (predominantly) powered by white, anaerobic muscle [40, 41], and the periodic acceleration/deceleration requires a more elaborate hydrodynamic model, the analysis of such swimming motion is outside the scope of this work.

In the following, we discuss the three main conclusions that can be drawn from our results. These conclusions provide a new insight into swimming energetics or challenge the established understanding of it. We primarily focus on the nature of energetic quantities and the relationships between them, as we have shown in the previous section that the absolute values of our results generally match the empirical measurements.

### Conclusion 1. The muscle efficiency and power output are not maximized in optimal swimmers

Swimming at the maximum sustained speed *U* was often related to the condition of producing the maximum power per muscle mass *ψ* [8, 42]. Later work-loop studies offered indications that this might not be the case [6, 7, 43], but the issue is still not conclusively resolved [15, 44]. Similarly, minimum-COT swimming was postulated to be related to the maximum of hydrodynamic efficiency [45, 46] or of muscle efficiency [3, 6]. Our results suggest, however, that neither of these conjectures may hold for *U*-optimal and COT-optimal swimmers.

Consider *U*-optimal swimmers first. A swimmer is increasing its swimming speed by increasing the tail-beat frequency *ω*. As the contraction velocity amplitude $\hat{v}$ is proportional to *ω* (Eq (2)), *U*-optimal swimmers strive to achieve as high $\hat{v}$ as possible. The maximum value of $\hat{v}$ that a swimmer can achieve is limited by the ability of its muscles to produce force. For a given $\hat{v}$, the maximum force can be exerted if the contraction velocity and the required muscle force are in phase (pure power mode, Φ = 0). In swimming, the hydrodynamic forces increase with $\hat{v}$ (increase in *ω* and *U*), while the maximum muscle force is a decreasing function of $\hat{v}$. Thus, *the maximum sustained swimming speed* (*U*)_*U*_ *is governed by the maximum amplitude* $\tilde{v}$ *of the contraction velocity, at which the balance of muscle and hydrodynamic forces is possible with a fully employed muscle* $\left(\hat{\mu }=1\right)$ *operating in the pure power mode* (Φ = 0). As long as the muscles can provide the required force at the given $\tilde{v}$, the average power production (or consumption) is irrelevant for sustained aerobic swimming because it can be continuously supplied. For realistic muscle fibers, our results show that the maximum value of ${\hat{v}}_{r}$ where this balance holds is below ${\tilde{v}}_{r}^{\eta_{m}}$ (and, thus, below ${\tilde{v}}_{r}^{P}$) for *U*-optimal swimmers, Fig 6C, so the muscles are operating in the monotonically increasing region of $\text{max}(\psi )–{\hat{v}}_{r}$ and $\eta_{m}–{\hat{v}}_{r}$ relationships (Fig 2G and 2H). As a consequence, all *U*-optimal swimmers have the highest total power output *P* and muscle efficiency *η*_*M*_ among the optimal populations, but they do not reach the maximum attainable values. Swimmers with near-maximum power output have been empirically observed in nature; for example, carp swim with maximum sustained speed by contracting their red muscle fibers with slightly lower velocity than ${\tilde{v}}_{r}^{P}$ [18]. The contraction velocities that result in near-maximum power output and efficiency that were found in these fish were the likely reason for relating the maximum sustained swimming speed with the maximum of power production.

**Fig 8:**
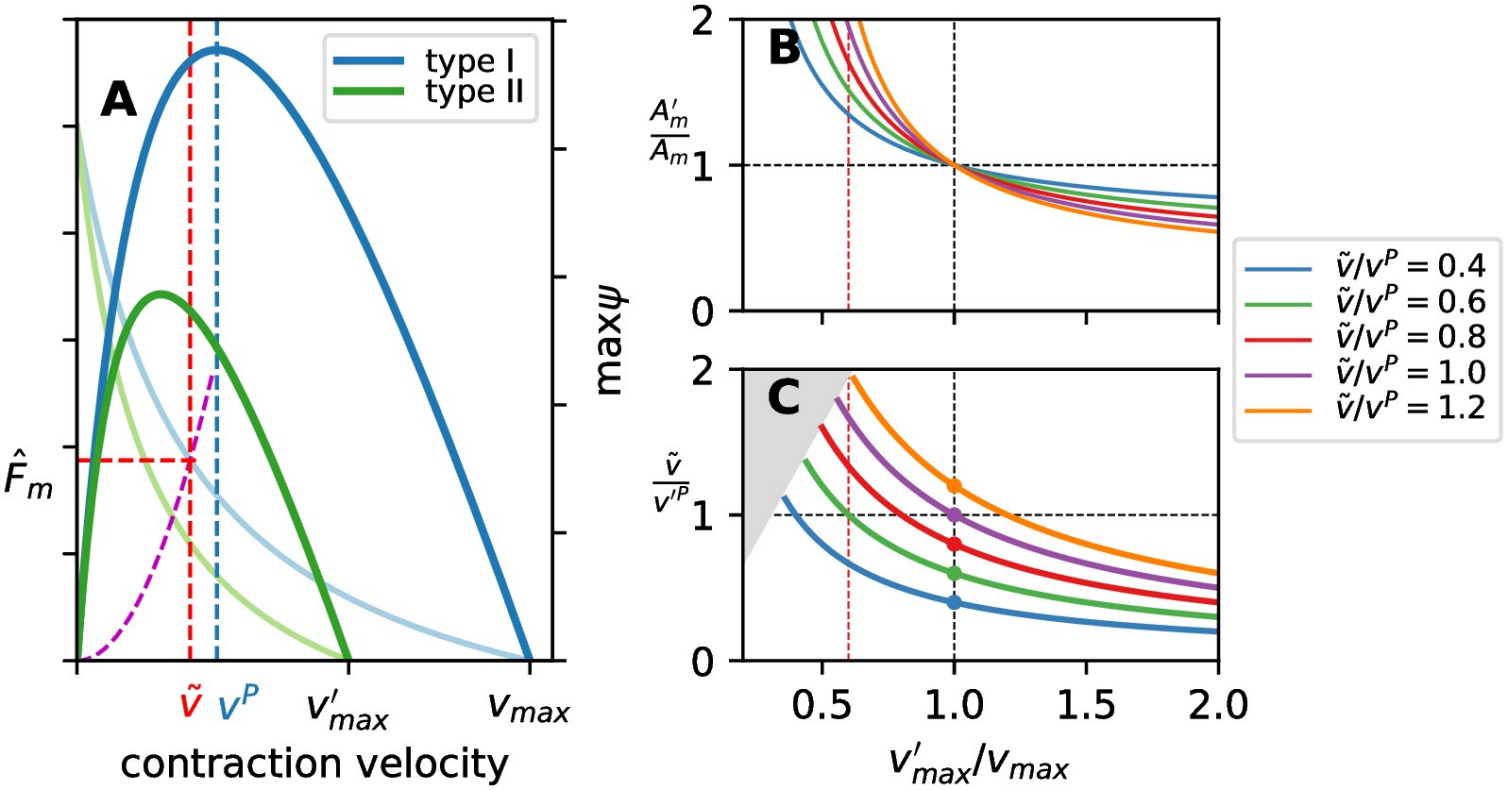
Effect of changing maximum contraction velocity *v*_max_. Consider fiber types I and II with maximum contraction velocities *v*_max_ and $v_{\text{max}}^{\prime }$ and the corresponding peak-power-output velocities *v*^*P*^ and *v*′^*P*^. (A) The muscle force amplitude ${\hat{F}}_{m}$ (thin lines) and the maximum mechanical power output per muscle mass max(*ψ*) (thick lines) as a function of the amplitude of contraction velocity $\hat{v}$ for fiber types I (blue) and II (green). The required force to overcome hydrodynamic loading is marked in dashed magenta line. The operating amplitude of contraction velocity $\tilde{v}$ and the required amplitude of muscle force (to balance the hydrodynamic loading) are marked in dashed red lines. For B and C, consider a range of $v_{\text{max}}^{\prime }$ for fiber II and corresponding *v*′^*P*^. The line colors correspond to the different operating contraction velocities $\tilde{v}$ (relative to fiber I). (B) The required change in the cross-section area $A_{m}^{\prime }$ of a muscle of type-II fibers to achieve the same muscle force ${\hat{F}}_{m}$. (C) The ratio between $\tilde{v}$ and *v*′^*P*^ if type-II fibers are used. For $\tilde{v}/{v^{\prime }}^{P}>1$, the muscle is operating at contraction velocities larger than that for maximum *ψ*. The gray region marks the unphysical cases ($\tilde{v}>v_{\text{max}}^{\prime }$).

Could a *U*-optimal organism swimming at (*U*)_*U*_ operate its muscles in a regime far from that where the power production per muscle mass *ψ* (or efficiency *η*_*m*_) is maximized? Generally, the answer is yes. To illustrate, consider the effect of replacing the existing muscle fibers with slower ones, i.e. those whose maximum contraction velocity $v_{\text{max}}^{\prime }$ is lower than *v*_max_ of the presently selected fibers; the corresponding dimensional peak-power-output contraction velocities are $v^{P}\equiv {\tilde{v}}_{r}^{P}\times v_{\text{max}}$ and ${v^{\prime }}^{P}\equiv {\tilde{v}}_{r}^{P}\times v_{\text{max}}^{\prime }$. In order for a swimmer to achieve the same swimming speed (*U*)_*U*_ when powered by different muscle fibers, the force provided by the muscles at every cross section has to remain the same for the given amplitude of contraction velocity $\tilde{v}$, regardless of the change in muscle fibers, Fig 8A. At a characteristic cross section, in order produce the required force with slower fibers $v_{\text{max}}^{\prime }<v_{\text{max}}$, the muscle cross section area has to increase ($A_{m}^{\prime }>A_{m}$), Fig 8B. Depending on the contraction velocity $\tilde{v}$ relative to the *v*^*P*^ of the original fibers and the relative change in $v_{\text{max}}^{\prime }$, the resultant operating regime can be significantly sub-optimal, from the standpoint of max *ψ* (the total power output $\bar{p}$ remains constant). The operating regime can even be in the monotonically decreasing range of the $\text{max}(\psi )–{\hat{v}}_{r}$ relationship, i.e. for $\tilde{v}>{v^{\prime }}^{P}$, Fig 8C. This provides further evidence that maximizing *ψ* is decoupled from maximizing *U* because, in this regime, increasing *U* (i.e. increasing ${\hat{v}}_{r}$) *decreases* *ψ*. The same holds for muscle efficiency *η*_*m*_. Such a condition is not only theoretically possible, but it also seems realistic. For example, to maintain the same optimal (*U*)_*U*_ using 60% slower muscle fibers (in our case this means $v_{\text{max}}^{\prime }=3\;\text{lengths}/\text{s}$), a characteristic cross section originally operating with ${\left({\hat{v}}_{r}\right)}_{U}\approx 0.24$, i.e. $\tilde{v}/v^{P}\approx 0.8$, will now operate with ${\hat{v}}_{r}^{\prime }>{\tilde{v}}_{r}^{P}$ and will require less than twice as large muscle cross section to achieve this, Fig 8B and 8C.

Our results also show that the departure from the established conjectures is perhaps even more striking for COT-optimal swimmers. Despite consuming the least amount of energy, COT-optimal swimmers exhibit the *minimum* muscle efficiency *η*_*M*_ among the optimal populations Π, Fig 5B. This, however, is no surprise. The reason for this behavior lies in the fact that COT-optimal swimmers achieve the lowest *U* among the optimal populations, as COT and *U* are conflicting objectives. As $U\propto {\hat{v}}_{r}$, they also swim with the lowest ${\hat{v}}_{r}$ among Π (see Fig 6C and [30]). As *η*_*m*_ is monotonically increasing function for ${\hat{v}}_{r}<{\tilde{v}}_{r}^{\eta_{m}}$, Fig 2G and 2H, for a characteristic cross section it will generally be the lowest for COT-optimal swimmers. As a result, COT-optimal swimmers also exhibit the lowest *η*_*M*_ among the optimal populations. More importantly, this illustrates that efficiencies, being a ratio of powers, are not a good indicator of optimality of COT when neither input nor output powers are prescribed, as is the case in this work—or, in fact, in nature. Even more questionable is correlating the minimum of COT primarily with a maximum of hydrodynamic efficiency [45, 46] (usually related to the flow structure of oscillating foils [47, 48]), while ignoring the underlying energy consumption of the prime mover. Our results show that the hydrodynamic efficiency is neither consistently maximum nor minimum among the optimal populations Π(*m*).

### Conclusion 2. The muscle efficiency decreases with swimmer size

In contrast to the increase in efficiency with size in flying and running animals [17], our results indicate that the same is not true in swimming. While larger organisms are generally better than the smaller ones in terms of COT as it is decreasing with body size for all organisms and all types of locomotion, the muscle efficiency *η*_*M*_ is consistently decreasing with body size, Fig 5A and 5B. As a result, the total efficiency *η*_*T*_ also decreases with *m* (for (*η*_*T*_)_COT_), or is roughly constant at best (say for (*η*_*T*_)_*U*_). To summarize, despite the decrease in the cost of transport COT with the increase in *m*, the efficiency of converting the metabolic energy into work is, surprisingly, decreasing.

The decrease in *η*_*M*_ with *m* stems from the fact that ${\left({\hat{v}}_{r}\right)}_{\text{opt}}$ is decreasing with *m*, (Fig 6C; also see Supplementary Information of [30]), thus lowering *η*_*m*_ along the body (see Fig 2H). Here, (⋅)_opt_ is a value corresponding to either *U*-optimal or COT-optimal organisms. The decrease in ${\left({\hat{v}}_{r}\right)}_{\text{opt}}$ is mainly driven by the decrease in (*ω*)_opt_ with *m* as ${\left({\hat{v}}_{r}\right)}_{\text{opt}}\propto {\left(\omega \right)}_{\text{opt}}\times {\left(B/L\right)}_{\text{opt}}\times {\left(h_{T}/L\right)}_{\text{opt}}$, and (*B*/*L*)_opt_ × (*h*_*T*_/*L*)_opt_ does not show appreciable scaling with *m* (see [30]). From a broader evolutionary perspective, it is still beneficial to be larger, since COT is a much more important energetic measure than efficiency [2, 17].

### Conclusion 3. Disparate longitudinal power output distributions can be reconciled by relating them to different optimization objectives

Our results indicate that both anterior-dominant and posterior-dominant longitudinal distributions of the average power output $\bar{p}\left(x\right)$ are possible in optimal swimmers, depending on which objective is optimized, Fig 6. This offers an explanation for the conflicting reports [6, 7, 11, 13–16, 49] on the character of $\bar{p}\left(x\right)$.

The longitudinal distribution of the average mechanical power output $\bar{p}\left(x\right)$ of *U*-optimal organisms across the scales is consistently maximum in the anterior part of the body, accompanied by nearly maximum muscle efficiency *η*_*m*_(*x*) in that region (except for the largest organisms). Such results are very similar to those obtained from mathematical modeling of red muscles [11] and empirical measurements [12, 49]. To achieve high power output, the muscles are almost fully employed (${\left(\hat{\mu }\left(x\right)\right)}_{U}\approx 1$) and the phase lag (Φ(*x*))_*U*_ is predominantly small, Fig 7. Close-to-optimal efficiency of power production (*η*_*m*_)_*U*_ is achieved for smaller organisms since ${\left({\hat{v}}_{r}\left(x\right)\right)}_{U}$ is close to ${\tilde{v}}_{r}^{\eta_{m}}$ in the anterior region, Fig 6C. In contrast, the anterior muscles of COT-optimal organism barely produce any power (${\left(\hat{\mu }\right)}_{\text{COT}}⪡1$, (Φ(*x*))_COT_ ≈ 90°), with most of the mechanical power coming from the posterior muscles, as empirically found [7, 44].

Thus, the COT-optimal and *U*-optimal swimmers exhibit distinctly different power production distributions, whose characteristics are almost independent of the change in the body shape and size. Both of these distributions seem to have counterparts in the real world. Note that a real organism might be neither *U*-optimal nor COT-optimal; it might be similar to some other optimal swimmer from the optimal population, i.e. the Pareto front, and its the performance could be a mix of the two extremes (e.g. see Fig 6).

While the previous conclusions provided a new perspective on and a potential resolution of some of the outstanding controversies in swimming energetics, our results also provide additional evidence for the well established theory that some fish use passive elements such as tendons instead of muscles in the caudal peduncle area to transfer power to the tail [14, 49, 50]. The results of the longitudinal distribution of the muscle activation fraction amplitude $\hat{\mu }\left(x\right)$ and phase lag Φ(*x*) support the notion that the muscles in the caudal peduncle can be effectively replaced by tendons. In general, the muscles in the tail section of optimal swimmers are considerably employed (*μ*(*x*) ≈ 1), but the phase lag Φ(*x*) ≳ 90° (Fig 7) indicates that the muscles are either doing no net work or that they are used for braking. Introducing additional passive elastic elements of increased stiffness (e.g. tendons) instead of muscles in this region would provide the necessary force at no metabolic energy cost as all the elastic energy can be recovered, thus lowering the energetic expense of swimming. (Such a swimmer can be modeled within our framework by either prescribing or optimizing the lengthwise distribution of stiffness and muscle cross-section area.) The muscles in the rest of the body would then be producing purely positive power (especially for smaller body sizes).

The results presented here reinforce the findings on the shape, motion and kinematic characteristics of the optimal populations [30], and the favorable comparison with a range of different empirical measurements lends further validity to our overall framework and to the muscle model in particular. This study shows that swimming at realistic speeds and energy expenditures is possible with the available muscle, thus providing another nail in the coffin of Gray’s paradox.

## Models

### Hydrodynamic model

We employ the classic Lighthill slender-body theory [31], which holds for small-amplitude undulatory motion and for high Reynolds numbers *Re*. Both of these assumptions are satisfied for the model swimmers we consider in this study. The distributed hydrodynamic lateral force on a slender body is $F_{L}\left(x,t\right)=D(m_{a}\left(x\right)\;D\hat{h}\left(x,t\right))$, where $D\equiv \partial_{t}+U\partial_{x}$ is the material derivative and *m*_*a*_(*x*) the cross-sectional added mass. Here, $\hat{h}\left(x,t\right)=h\left(x,t\right)+y_{0}\left(t\right)+x\;\varphi \left(t\right)$ is the total deflection of the body, composed of the undulatory motion *h*(*x*, *t*), the lateral recoil *y*_0_(*t*) and the angular recoil *φ*(*t*), Fig 1C. The unknown rigid-body recoil is determined from the lateral force and moment balance equations.

The balance of time-averaged in-line forces (forward pointing thrust $\bar{F_{T}}$ and backward drag force $\bar{F_{D}}$) determines the steady-swimming speed *U*. For Lighthill’s slender-body model, the average thrust can be obtained analytically [31]. The drag force, not being tractable within the potential flow framework, is expressed as $\bar{F_{D}}=0.5\rho U^{2}SC_{D}$, where *S* is the wetted surface of the body. The drag coefficient *C*_*D*_ is determined from an empirical formula [51]

$$\begin{array}{c} \begin{array}{cc} C_{D}\left(Re,\text{shape}\right) & =C_{f}\left(Re\right)(1+1.5D_{L}^{1.5}+7.0D_{L}^{3})\\ C_{f}\left(Re\right) & =\{\begin{array}{cc} 1.33\;Re^{-0.5}, & Re\leq Re_{cr}\\ 0.072\;Re^{-0.2}, & Re>Re_{cr} \end{array}, \end{array} \end{array}$$ (8)

where *Re*_*cr*_ = 5.0 ⋅ 10^5^ is the critical Reynolds number and *D*_*L*_ ≡ *D*/*L*.

The total power delivered to the fluid is $P_{H}=I[F_{L}\left(x,t\right)\partial_{t}\hat{h}]$, where $I[\cdot ]\equiv 1/T\int_{0}^{T}\int_{0}^{L}\left(\cdot \right)\;dx\;dt$. The hydrodynamic efficiency is then $\eta_{H}\equiv \bar{F_{T}}U/P_{H}$.

#### Note on the validity of the hydrodynamic model

Today, when state-of-the-art computational fluid dynamics (CFD) studies offer an unprecedented look into the flow around a swimming body [20–22, 52], one could question the validity of using a simple, slender-body potential flow hydrodynamic model [31] and empirical formulas for drag that we use in this work. Due to the large range of Reynolds numbers *Re* and the extensive computational scope of this study, we seek fast models that capture the most important flow characteristics (such as inertial effects and the transition from the laminar flow to turbulent) in the given *Re* range.

To obtain the results presented in this work required *O*(10^7^) simulations of different swimmers using different undulatory motion, over a large range of Reynolds numbers (*Re* ∼ 10^4^–10^8^). As a comparison, to perform a simulation for a single swimmer and a given undulatory motion using CFD methods would require tens, if not hundreds, of processor-hours, rendering the overall computational study based on CFD methods infeasible. Furthermore, we are mostly interested in the nature and scaling of the salient energetic quantities with the increase in size (i.e. *Re*), rather than on the specific features of the flow around a particular swimmer. As *Re* increases, CFD studies become computationally infeasible (currently limited to *Re* up to *O*(10^4^)), while the potential flow models become more and more valid (the flow around a swimmer is becoming increasingly more similar to potential flow). This indicates that the scaling of hydrodynamic forces with the increase in size (i.e. *Re*) is well captured by potential flow models. Coupling a CFD model of a particular swimmer with our muscle model could offer a more realistic view of the swimming energetics for that particular case and serve as an important check of the results presented here (e.g. of the hydrodynamic efficiency *η*_*H*_, Fig 5B). However, we believe that the overall energetic traits would follow those described in this work.

The Lighthill’s potential flow model [31] we use introduces two further assumptions—the body needs to be slender and the amplitude of the motion small. Lighthill also developed a large-amplitude motion elongated-body model that is more generally valid [53]. However, since the resulting motion amplitude of all the swimmers in this work is small, this more general method would not produce significantly different results than those presented here, but it would result in an increased computational cost. Similarly, (semi-)analytic potential flow models of swimming that are valid for a certain types of body shapes come at an increased computational cost (e.g. Chopra’s theory for lunate tail propulsion [54, 55] would only be valid for body shapes with a pronounced caudal peduncle), while not offering the generality needed for an optimization study where different shapes are involved. Thus, despite the simplicity of the Lighthill’s model, the conclusions of this work should hold as they mainly refer to the nature and scaling of the swimming energetics, rather than on the particular detailed features.

### Structural model

The balance of the distributed internal forces and external hydrodynamic forces acting on a swimming body is modeled using the Euler-Bernoulli beam equation [25]

$$\begin{array}{c} \rho A\left(x\right)\frac{\partial^{2}\hat{h}}{\partial t^{2}}+\frac{\partial^{2}}{\partial x^{2}}(EI\left(x\right)\frac{\partial^{2}\hat{h}}{\partial x^{2}})+\frac{\partial^{2}}{\partial x^{2}}(\nu_{b}I\left(x\right)\frac{\partial^{3}\hat{h}}{\partial t\partial x^{2}})+F_{L}=-\frac{\partial^{2}M}{\partial x^{2}}\;, \end{array}$$ (9)

where *I*(*x*) is the sectional moment of inertia. The above terms, corresponding respectively to forces due to inertial, elastic, visco-elastic, and hydrodynamic effects are all balanced by the bending moment *M* produced by muscles. Aggregate Young’s modulus *E* and visco-elastic coefficient *ν*_*b*_ (which accounts for visco-elastic losses) correspond to the combined contribution at each cross section along the length from all the passive elements where elastic energy is stored and dissipated during bending: elasticity and visco-elasticity of the spine, the skin, the white muscle, and the inactive part of red muscles (assuming that the morphology of the model swimmers is equivalent to that of fish).

The sectional bending moment *M*(*x*, *t*) is directly obtained from (9) for a given $\hat{h}\left(x,t\right)$ which satisfies the necessary boundary conditions ($\partial_{xx}\hat{h}=0$, $\partial_{xxx}\hat{h}=0$) at both *x* = 0 and *x* = *L*. The total power lost in overcoming internal visco-elastic losses can be expressed as $P_{V}=I[\nu_{b}I\left(x\right){\left(\partial_{xxt}\hat{h}\right)}^{2}]$. Using the decomposition of the total power output into *P* = *P*_*H*_ + *P*_*V*_, we obtain the internal efficiency *η*_*I*_ as

$$\begin{array}{c} \eta_{I}\equiv \frac{P_{H}}{P}=\frac{1}{1+P_{V}/P_{H}}\;. \end{array}$$ (10)

### Hill’s equations

We assume that the contraction force *F*_*f*_ of a muscle fiber is a function of fiber contraction speed *v*(*x*, *t*) only; it is described by Hill’s model [33] as

$$\begin{array}{c} \frac{F_{f}}{F_{0}}=\{\begin{array}{cc} 1.8-0.8\frac{1+v_{r}}{1-7.56\;G\;v_{r}}, & -1\leq v_{r}<0\\ \frac{1-v_{r}}{1+G\;v_{r}}, & 0\leq v_{r}\leq 1 \end{array}\;. \end{array}$$ (11)

We take *G* = 4, following [33]. We assume that the metabolic power consumed per fiber *Q*_*f*_ is also a function of *v* only, given by Hill’s model [33]

$$\begin{array}{c} \frac{Q_{f}}{F_{0}\;v_{max}}=\{\begin{array}{cc} 0.01-0.11v_{r}+0.06\;\text{exp}\left(23v_{r}\right), & -1\leq v_{r}<0\\ 0.23-0.16\;\text{exp}(-8v_{r}), & 0\leq v_{r}\leq 1 \end{array}\;. \end{array}$$ (12)

### Optimization setup

The body shape and motion are parameterized in such a way to allow the description of very different distributions with a relatively small number of parameters, while satisfying the motion feasibility and shape integrity conditions. The shape is parameterized by using the coefficients of a series of shape functions designed specifically for this purpose. The motion envelope is parameterized using the coefficients of a Chebyshev series. The body wavelength λ_*b*_ = *L* is kept constant. As we do not focus on shape or motion aspects of optimal organisms in this text, we refer the reader to [30] for more details.

Optimization is conducted for organisms of mass *m* = *a*10^*b*^, with log *a* = 0, 1/4, 1/2, 3/4 and *b* = −3, …, 6. We use a multi-objective covariance matrix adaptation evolutionary strategy (MO-CMA-ES), with default parameters. MO-CMA-ES is a stochastic, derivative-free optimization method where the new candidate solutions are sampled from a multi-dimensional normal distribution, whose covariance matrix is evolved through optimization iterations [36, 56, 57]. The resulting algorithm is quasi parameter-free. For every *m*, an initial randomly generated feasible population of 500 individuals is evolved through 500 generations. The optimization converges in all cases, and the bounds imposed on the variables are never active in the final population.

### Assumed body/muscle/fluid properties

For simplicity, in all our calculations muscle and tissue properties are taken as length and size independent, but characteristic for fish (red fiber isometric force *F*_0_ = 150 kN/m^2^, *v*_max_ = 5 lengths/s [58], *E* = 10^5^ N/m^2^, *ν*_*b*_ = 10^4^ m^2^/s [25, 26, 29], *μ*_0_ = 0.1 [16]). The standard metabolic rate used here is *P*_*s*_ = 0.1327*m*^0.80^ [W] [59]. Fresh water properties are used throughout (*ρ* = 10^3^ kg/m^3^, *ν* = 10^−6^ m^2^/s). The missing mass measurements in the empirical data presented in Figs 4B and 5A are supplied from *m* − *L* allometric expression (*m* = 12.62*L*^3.11^) obtained from fish data [4].
